# A dynamic traffic signal scheduling system based on improved greedy algorithm

**DOI:** 10.1371/journal.pone.0298417

**Published:** 2024-03-15

**Authors:** Guangling Sun, Rui Qi, Yulong Liu, Feng Xu

**Affiliations:** 1 School of Electronic and Information Engineering, Anhui Jianzhu University, Hefei, China; 2 Intelligent Interconnected Systems Laboratory of Anhui Province, Hefei University of Technology, Hefei, China; Southwest Jiaotong University, CHINA

## Abstract

Urbanization has led to accelerated traffic congestion, posing a significant obstacle to urban development. Traditional traffic signal scheduling methods are often inefficient and cumbersome, resulting in unnecessary waiting times for vehicles and pedestrians, exacerbating the traffic situation. To address this issue, this article proposes a dynamic traffic signal scheduling system based on an improved greedy algorithm. Unlike conventional approaches, we introduce a reward function and a cost model to ensure fair scheduling plans. A constraint function is also established, and the traffic signal scheduling is iterated through the feasible matrix using the greedy algorithm to simplify the decision-making process and enhance solution efficiency. Moreover, an emergency module is integrated to prioritize special emergency vehicles, reducing their response time during emergencies. To validate the effectiveness of our dynamic traffic signal scheduling system, we conducted simulation experiments using the Simulation of Urban Mobility (SUMO) traffic simulation suite and the SUMO traffic control interface Traci. The results indicate that our system significantly improves intersection throughput and adapts well to various traffic conditions, effectively resolving urban traffic congestion while ensuring fair scheduling plans.

## Introduction

### Background and purpose

The number of motor vehicles is steadily increasing, particularly during peak commuting periods, leading to a rise in urban congestion. This congestion not only causes inconvenience to travelers but also contributes to increased fuel consumption and higher emissions of car exhaust [1]. Such air pollution poses serious health risks to residents [2] and conflicts with the principles of ecological civilization construction.

Conventional traffic light scheduling, with a fixed duration and fixed sequence, often results in the frustrating occurrence of “green light for vehicles-free” reducing the road capacity and wasting valuable time for people. This phenomenon, commonly known as “empty green lights” exacerbates traffic congestion by allowing traffic signals to turn green even when no vehicles are waiting to proceed. Consequently, valuable green light time is squandered, exacerbating the overall traffic flow inefficiency. Additionally, “empty green lights” can lead to unnecessary casualties as emergency vehicles, such as ambulances and fire trucks, are unable to reach their destinations promptly.

Given the prevalence of congested roads, traffic police frequently resort to manually adjusting the duration of traffic signals. However, this approach is slow to respond and inefficient. Consequently, traditional traffic light scheduling is no longer suitable for today’s diverse traffic conditions.

In recent years, the rapid development of big data and artificial intelligence has significantly improved the efficiency and stability of road traffic signal scheduling systems. Currently, the primary traffic light scheduling system still relies on extending and shortening the signal timing based on fixed sequences. However, this approach often leads to the occurrence of “empty green lights” wasting valuable traffic resources. Literature review

This article proposes a dynamic signal light scheduling system that utilizes an improved greedy algorithm to change the fixed sequence. By employing image recognition and signal transmission [3,4], traffic conditions at intersections are collected, and the optimization of traffic conditions is formulated as a problem. The problem is then divided into several time intervals, and reward, cost, and constraint functions are defined [5,6]. Based on whether the routes conflict, the best scheduling plan is selected. Furthermore, this system fully considers the actions of special emergency vehicles, such as ambulances and fire trucks, to ensure their timely response.

To validate the effectiveness of the dynamic signal light scheduling system in simulating road traffic flow, the Simulation of Urban Mobility (SUMO) traffic simulation suite, along with the SUMO Traffic Control Interface (Traci), is utilized for simulation and evaluation.

The paper is structured as follows: Section II describes the current state of research in signal scheduling. Section III describes the scheduling environment and the simulation model used for algorithm verification. Section IV presents the principles of the modified greedy algorithm for the dynamic signal light scheduling system with changing fixed order. Section V discusses the results and provides an analysis. Section VI outlines future work and potential developments. Finally, Section VII offers a summary.

## Literature review

### Signal dispatch

Road traffic lights have evolved significantly, moving away from the traditional fixed signal timing mode to embrace the more intelligent signal timing mode. In recent years, artificial intelligence (AI) has emerged as a promising solution for traffic signal control. Researchers have explored various AI-based approaches, such as fuzzy theory [7,8], fuzzy neural networks [9], and fuzzy control models [10,11], to design signal control schemes.

Additionally, reinforcement learning has been applied to learn optimal signal control strategies [12–14]. The advanced Reinforced AIM (adv.RAIM) system, employing end-to-end Multi-Agent Deep Reinforcement Learning (MADRL), presents a novel paradigm for Autonomous Intersection Management (AIM) [15]. Its notable advantages over traditional traffic light control methods underscore its potential to revolutionize signal scheduling systems, offering improved adaptability and efficiency in traffic management. Van Der Pol investigated traffic signal coordination using a DQN, employing a testbed with a right-angle intersection and turn prohibitions. Simulation of Urban MObility (SUMO) was used for simulation, utilizing vehicle location from image data for the state, and selecting signal combinations as actions. The reward, a weighted average, considered vehicle delay, waiting time, stops, and signal changes [16]. Genders and Razavi optimized traffic signals using a DQN with a deep CNN. The testbed had a right-angle intersection with four lanes in each approach. SUMO simulated the scenario, extracting vehicle positions from images. The reward was based on the change in cumulative vehicle delay [17]. Evolutionary algorithms (EAs), including genetic algorithms and particle swarm optimization [18–23], have also been utilized for static timing optimization problems.

As shown in Table 1, several traffic signal control systems, such as TRANSYT [24,25] and SCOOT [26] in the United Kingdom, SCATS in Australia [25,27], RHODES and OPAC in the United States [28,29], the CRONOS system in France [30], and the SPOT system in Italy [31], have been studied and developed worldwide.

**Table 1:** Comparison of traffic signal control between modern and contemporary times [32].

Control system	Area	Time	Principle	Advantages and Disadvantages
TRANSYT SYSTEM	United Kingdom	1969	Using the hill climbing algorithm to optimize the green time ratio and phase difference for timing settings of the signal system, under stable traffic flow conditions.	Advantage: A timed operating system is easy to use and does not require complex operations. Disadvantage: The traffic signal cycle must be consistent, and the implementation environment must be too idealized.
SCOOT SYSTEM	United Kingdom	1979	Installing vehicle detectors at intersections can allow for real-time acquisition of vehicle entry and exit times and intervals on the road. This method can be used to adjust the timing of traffic signals, thereby reducing the average waiting time at the intersection.	Advantage: The system has the characteristics of fast response, strong stability, and low error rate. Disadvantage: Due to the high complexity and sudden changes in traffic, there may be some challenges.
SCATS SYSTEM	Australia	1969 Around	Setting up vehicle detectors at the stopping points of intersections is an adaptive control scheme that optimizes timing plans by integrating system parameter information.	Advantage: It does not require complex traffic models and has comprehensiveness. Disadvantage: The solution design is limited, and the system reliability is low.
RHODES SYSTEM	United States	1996	Develop a transportation prediction model to forecast and simulate traffic conditions in advance, to coordinate the timing and phasing of signals. Simultaneously, optimize scheduling by monitoring traffic flow in all directions.	Advantage: This solution has strong predictive ability and stability. Disadvantage: In the case of high traffic volume, prediction becomes more difficult and complex.
OPAC SYSTEM	United States	1983	Coordinating the road traffic signal system in a decentralized intersection network can alleviate information overload in centralized networks while taking into account the dynamic environment of traffic patterns.	Advantage: The signal controller has excellent coordination and processing capabilities. Disadvantage: The communication rate is low, and the control algorithm is more complex.
CRONOS SYSTEM	France	1996	Using heuristic methods to find the minimum local delay based on intersection queue length and vehicle occupancy rate, to improve the control performance of real-time urban traffic control systems.	Advantage: Strong global optimization ability, which can significantly shorten the total delay time. Disadvantage: The implementation environment is complex, and the switching frequency is low in complex situations.
SPOT SYSTEM	Italy	1985	The city’s traffic optimization control system is a macro traffic model based on historical data, which implements a bus priority strategy by introducing the concept of weights.	Advantage: Based on historical data, it has good predictability; public transportation efficiency can be improved through bus priority strategies. Disadvantage: It requires sufficient collection and analysis of historical data, and the system implementation complexity is high.

According to the operational situation, traffic signal controllers can be classified into two categories: timing signal controllers and adaptive signal controllers [7]. Timing signal controllers have fixed cycle lengths and green light splitting, while adaptive signal controllers can be adjusted dynamically, making the latter more effective in traffic control [33]. Despite the advances in various methods, designing effective and efficient urban traffic signal control systems still poses challenges.

### Discrimination of research

Adaptive traffic signal control systems employ two primary strategies to optimize traffic flow at intersections and road segments: adjusting fixed signal durations and altering the fixed sequence of signals. Fixed-duration systems, such as traditional traffic signal control, are suitable for situations where traffic flow remains relatively constant. These systems allocate green signal time based on preset intervals, providing stability but lacking adaptability to changing conditions. In contrast, adaptive systems like SCOOT and SCATS dynamically adjust signal durations and phase sequences in real-time, responding to the evolving traffic environment.

The advantages of fixed-duration systems lie in their simplicity and controllability, making them suitable for relatively stable traffic patterns. However, in urban areas with fluctuating and dynamic traffic volumes, adaptive systems demonstrate clear advantages. These systems optimize traffic flow by adjusting signal timings based on real-time data, potentially reducing congestion and enhancing overall commuting experiences. Nevertheless, they may entail higher technical support and maintenance costs.

Therefore, to strike a balance between cost considerations and scheduling efficiency, we are inclined to focus our research solely on altering fixed-sequence signal scheduling systems. This approach retains a degree of system simplicity and controllability while adapting to fluctuating traffic flow by flexibly adjusting the sequence of signal timings. Compared to fixed-duration systems, this method may offer enhanced performance in urban areas with variable and dynamic traffic volumes.

By concentrating on adjusting the fixed sequence of signals, we aim to reduce technical support and maintenance costs while preserving the flexibility of the scheduling system as much as possible. This balance may be particularly suitable for urban traffic management, where sensitivity to real-time changes is crucial, but economic considerations must be carefully weighed. Through an in-depth exploration of altering fixed-sequence signal scheduling systems, we hope to identify a cost-effective and flexible solution to meet the demands of urban traffic efficiently.

## Related work

### Scheduling environment and conditions

To control all signal lights at an intersection and achieve global traffic signal scheduling, it is crucial to detect the traffic flow in each lane accurately. For vehicle detection, we propose the installation of a camera in each direction on the traffic light pole to monitor and integrate real-time traffic flow at the intersection. However, for pedestrians, relying solely on cameras may not yield optimal results due to challenges such as overlapping pedestrians, greenery, and building obstructions. Therefore, we need to explore more effective detection methods for pedestrian flow.

Currently, we employ a pedestrian crosswalk button on the traffic light pole, which is a widely used approach in the United States [34]. This button serves multiple purposes: it facilitates traffic signal scheduling at intersections and ensures pedestrian safety while crossing the road. However, to accommodate special emergency vehicles, we propose equipping them with remote control devices that can communicate with emergency signal receivers installed in traffic signal lights, allowing for priority emergency scheduling. This measure aims to expedite the passage of emergency vehicles during critical situations.

### Simulation model building

SUMO is an excellent traffic simulation software widely used in traffic planning, management, and intelligent transportation systems [35,36]. It offers several advantages: 1) it is open source, allowing users to utilize, modify, and distribute it freely; 2) it is flexible and capable of simulating various road traffic scenarios, automatically adhering to the regulations of the “Traffic Safety Law of the People’s Republic of China” for vehicle acceleration and deceleration; 3) It provides a user-friendly interface, detailed documentation, Python API, and command-line tools, as well as various formats of simulation data for easy analysis and system validation; 4) it is extendable, allowing users to customize algorithms, models, and write plugins to enhance its functionality.

Based on these advantages, we selected the open-source traffic simulation software SUMO as the simulation model for our dynamic traffic signal scheduling system. We created a map featuring a four-way intersection with three lanes (left turn, right turn, and straight) and one pedestrian lane in each direction, resulting in a total of sixteen routes in the road network requiring traffic control.

**Fig 1:**
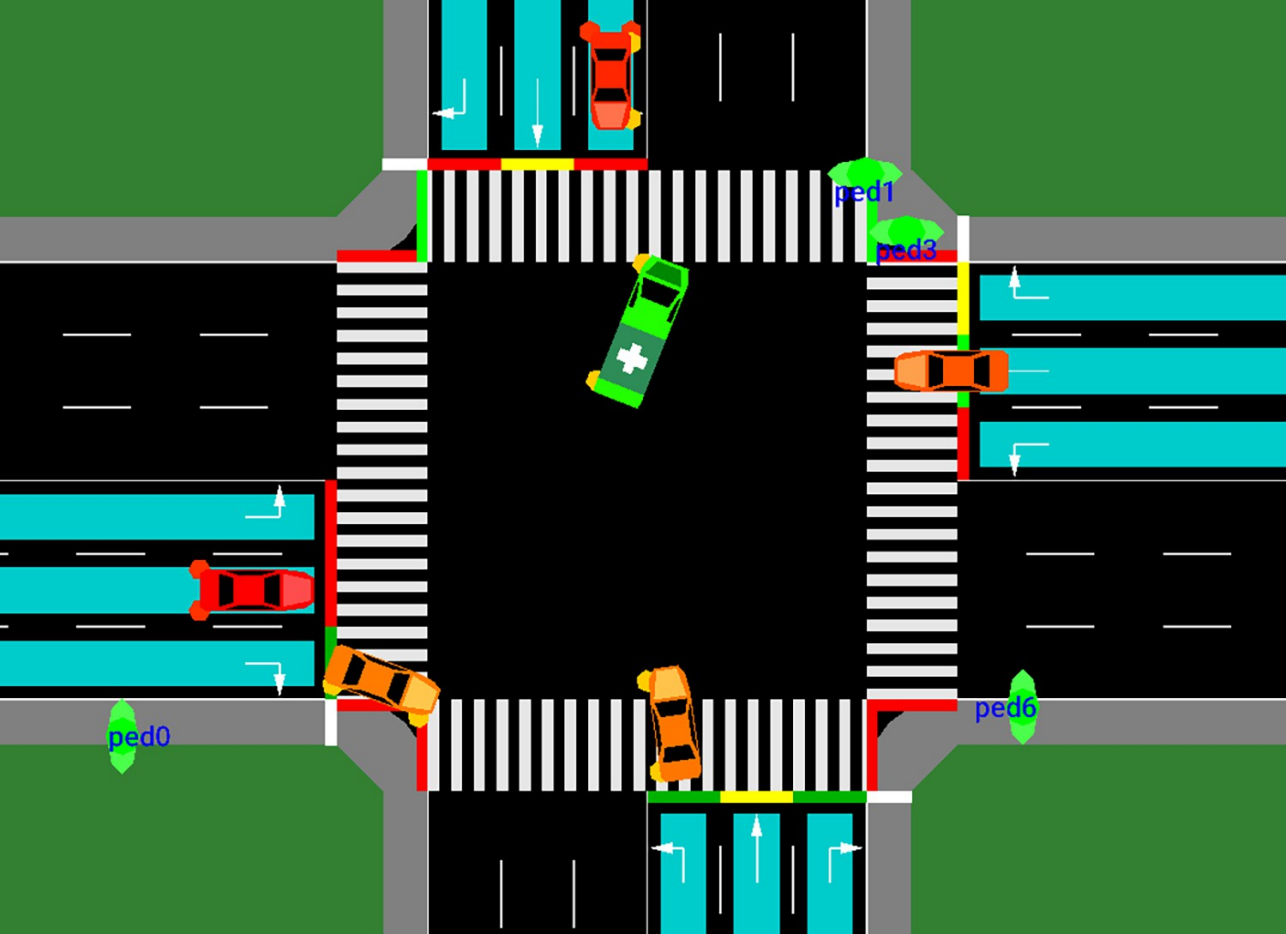
SUMO simulation of the intersection.

In Fig 1, the blue area represents the region where real intersection cameras detect vehicles. These cameras monitor this area to gather information about the presence and movement of vehicles, allowing for efficient traffic signal control. For pedestrians, the intersection itself provides pedestrian information, and their movements are monitored within the intersection area to ensure their safety.

As for special emergency vehicles, such as ambulances, they are designated with a cross displayed on their body above Fig 1. These vehicles have the characteristic of ignoring red lights, enabling them to pass through the intersection without stopping when necessary, ensuring a swift response during emergencies. Additionally, the green elliptical object in Fig 1 symbolizes a pedestrian, indicating their presence within the monitored area.

In SUMO, the default traffic signal control relies on traditional static scheduling. However, we utilize Traci as a third-party library for Python, which serves as an interface connecting Python scripts to the traffic simulation software SUMO. This integration allows us to access real-time parameters from the simulation and control it accordingly.

During each simulation, we gather vehicle and pedestrian information, which would typically be obtained from cameras and buttons in real-life scenarios, through Traci. Leveraging this data, we apply an improved greedy algorithm-based dynamic traffic signal control algorithm to generate the current optimal traffic signal control scheme. By using Traci and implementing the dynamic traffic signal control algorithm, we can effectively enhance the SUMO simulation to reflect real-world traffic conditions more accurately, enabling us to evaluate the performance of our proposed approach and optimize traffic signal timings based on the dynamic flow of vehicles and pedestrians.

## System principle

Dynamic time-sequencing optimization problems necessitate real-time adaptation to changing conditions, and one effective approach is through the application of greedy algorithms. Rooted in local optimization, these algorithms make sequential, myopic choices at each step, aiming to approximate the global optimum. This optimization challenge involves determining the most efficient arrangement of elements over time, considering the evolving nature of the system.

Building upon the broader application of greedy algorithms in optimization problems [37,38], our emphasis on dynamic time-sequencing optimization highlights the versatility of this approach. Recognized for their locally optimal decision-making, greedy algorithms play a pivotal role in resource-constrained scenarios, spanning time, space, and cost considerations. This adaptability proves especially valuable in domains like sensor scheduling, where these algorithms excel in minimizing estimation errors and enhancing overall system performance [39]. In the context of production scheduling, the Iterated Greedy Algorithm has proven efficient in resolving intricate problems [40]. Moreover, our focus on dynamic time-sequencing optimization resonates in domains such as wind turbine positioning, where the integration of the greedy algorithm with incremental calculation and iterative adjustments surpasses alternative methods, establishing itself as a highly effective strategy for optimal solutions [41]. This underscores the relevance and efficacy of the greedy algorithm in addressing a spectrum of optimization challenges.

To address the dynamic traffic light scheduling optimization problem, characterized by the imperative to minimize traffic congestion and waiting times while enhancing overall traffic efficiency, the application of the greedy algorithm proves instrumental [42]. Greedy algorithms, renowned for their ability to make immediate, locally optimal decisions, exhibit a fast execution that aligns seamlessly with the real-time demands of traffic management. Through the utilization of greedy algorithms in this context, our approach significantly improves traffic efficiency and reduces congestion, accomplishing the objectives of dynamic traffic light scheduling.

**Fig 2:**
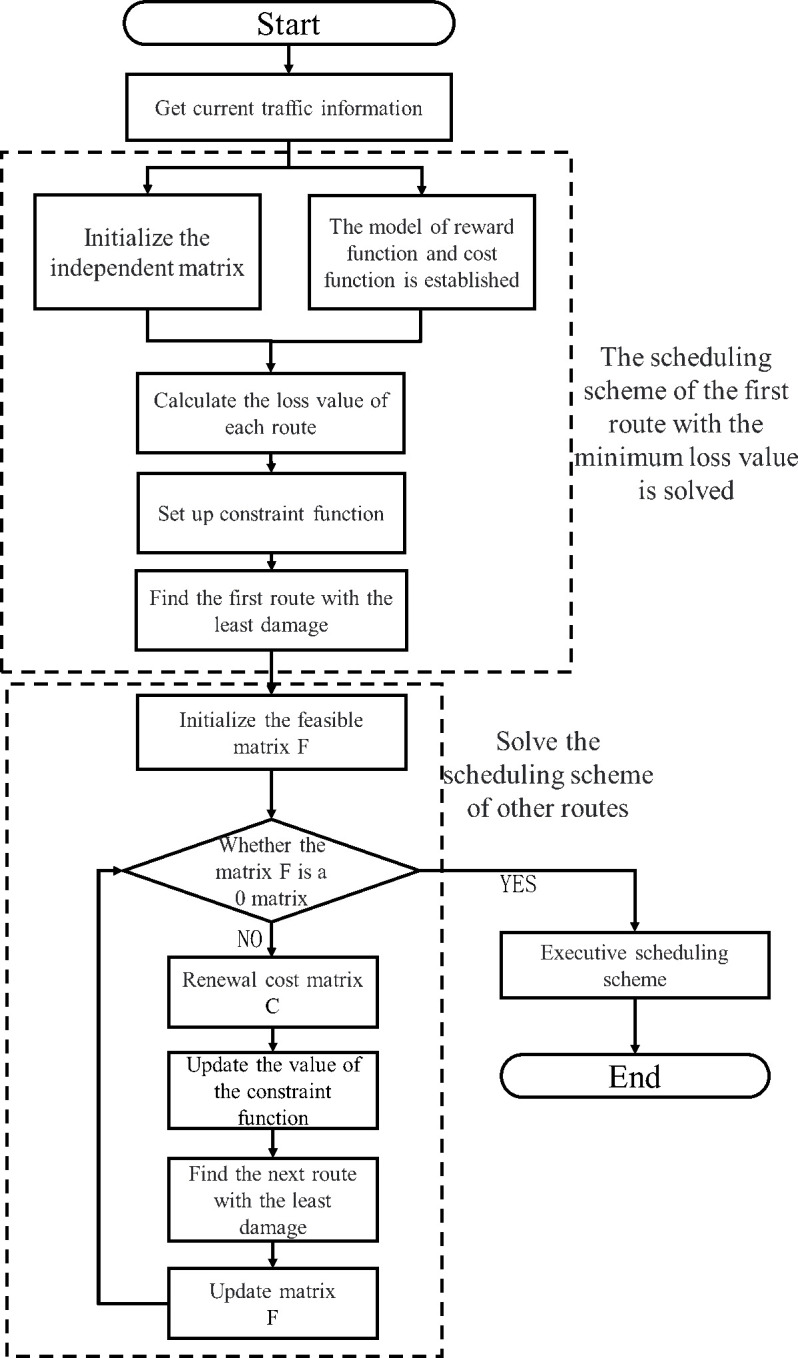
Structure diagram of dynamic signal light scheduling algorithm.

To further optimize traffic flow control, we propose introducing reward functions, cost functions, and constraint functions to design more efficient greedy algorithms. Specifically, we obtain information on the number of vehicles and pedestrians on the current road, establish reward and cost functions to calculate the total loss value of each route, and define constraint functions. The algorithm then selects the first route and iteratively schedules the signal lights through the feasible matrix, dynamically adjusting the signal light status on each route to optimize traffic flow. The utilization of greedy algorithms enables the division of the optimized traffic flow into several periods, simplifying the decision-making process and significantly improving solution efficiency. This approach facilitates the efficient and rapid transmission of dynamic signal light scheduling decisions to signal light devices, as depicted in Fig 2.

### Define the problem and subproblem breakdown

To find out the best signal scheduling scheme in the current time interval, we need the current traffic situation at the intersection, and we take the average waiting time of vehicles and pedestrians in the period as the current traffic situation.

We divide the optimization traffic flow problem into several time intervals and calculate the optimal scheduling scheme for each interval. Decomposing the problem into smaller intervals enhances the efficiency and accuracy of the solution, thereby maximizing traffic flow.

**Fig 3:**
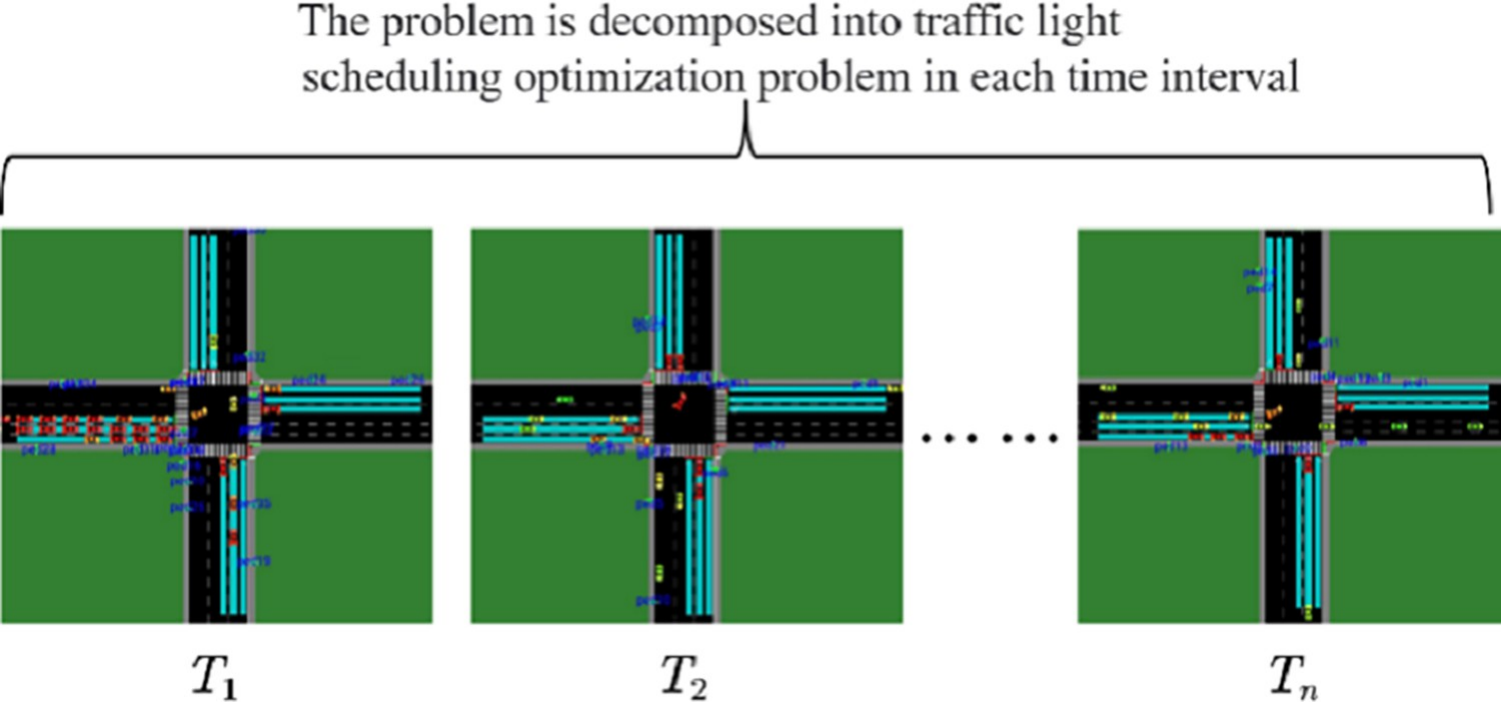
Decompose the problem.

The greedy strategy is designed to assign green lights to the direction with the highest vehicle traffic passing through the intersection at each time interval. The traffic light status of the intersection is determined based on the detected traffic flow status at each intersection (Fig 3).

### Introduce reward, cost, and constraint functions

Taking into full consideration the disadvantages of the local optimal solution caused by the greedy algorithm, the cost function can be employed to prevent a constant state of red lights in specific lanes, reduce the standard deviation of the average vehicle waiting time, and ensure fairness for both vehicles and pedestrians. The rules for setting the cost function are as follows:

$$P_{i}=\{\begin{array}{ll} 0 & No\;traffic\;on\;the\;road\\ 1 & The\;first\;traffic\;on\;the\;road\\ (P_{i-1}+1)P_{i-1} & otherwise \end{array}$$ (1)

Thereinto, *i* is the route *i* at an intersection, *P*_*i*_ is the generation value assigned to each route.

To determine the optimal state of the current traffic lights, we consider the reward value associated with allowing traffic on specific routes, which represents the benefit of permitting traffic flow on those roads. Set the reward function to be equal to the cost function, namely ***R***_***i***_ = ***P***_***i***_.

**Fig 4:**
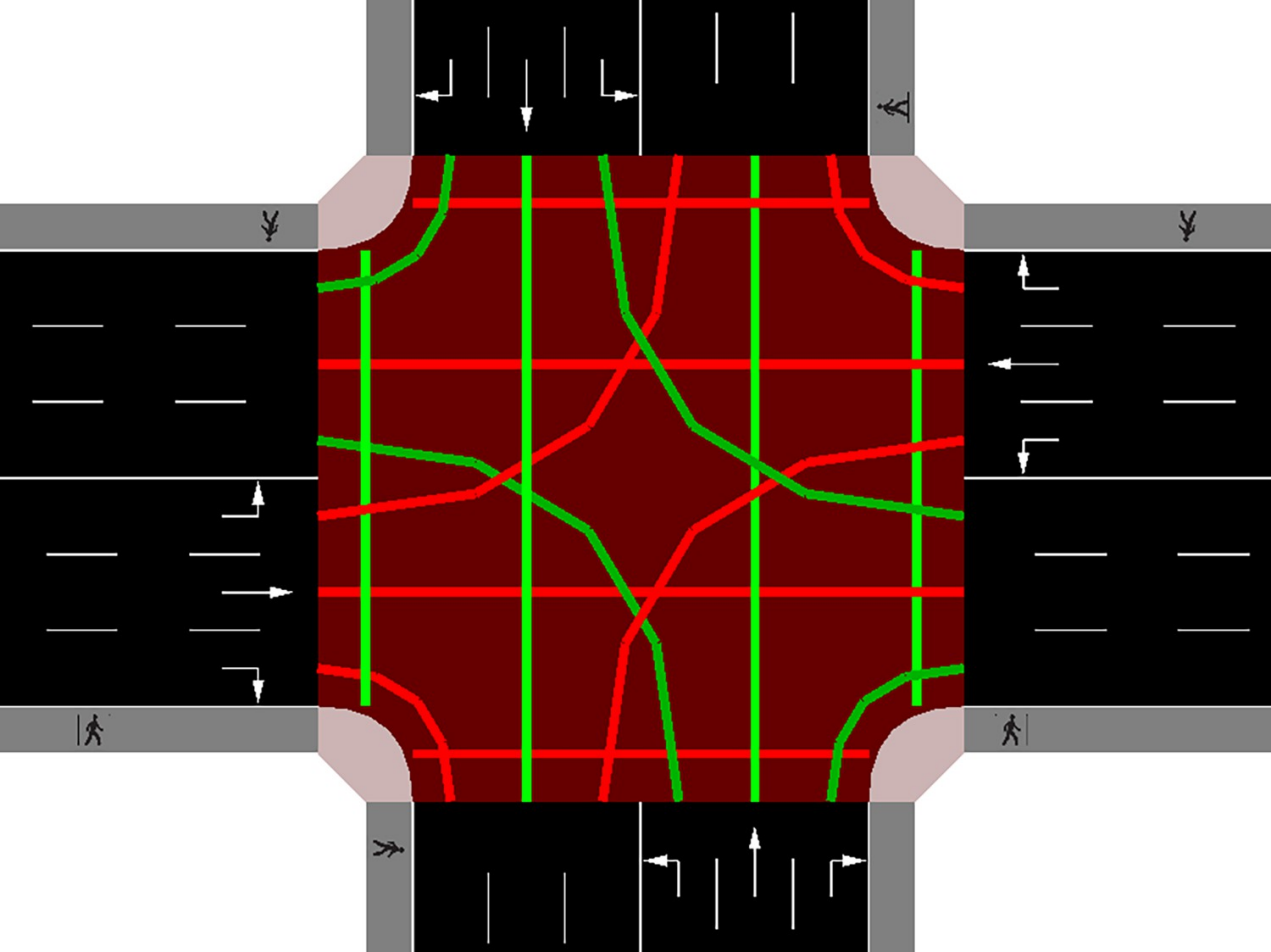
Route independence and conflict.

As shown in Fig 4, the intersection consists of four directions: east, west, south, and north, which are controlled by corresponding traffic lights, resulting in a total of sixteen routes. These routes encompass various movements, including West - Left, West - Direct, West - Right, West - Pedestrian, South - Left, South - Direct, South - Right, South - Pedestrian, East - Left, East - Direct, East - Right, East - Pedestrian, North - Left, North - Direct, North - Right, and North - Pedestrian. Among these sixteen routes, some are independent of each other, while others conflict with each other. To represent the relationship between routes, we define a 16*16 two-dimensional matrix A. If matrix[i][j] is 1, then route *i* is independent of route j. If matrix[i][j] is 0, then route *i* conflicts with route j. If matrix[i][j] is -1, then route *i* and route j are the same. Thus, we can initialize the independent matrix A according to the traffic rules.

For the independent matrix A, we need to transform it as follows:

$$\begin{array}{l} \;E[i][j]=A[i][j](1,\;-1\to 0,\;0\to 1)\\ \Rightarrow E=\left[\begin{array}{cccccccccccccccc} 0 & 0 & 0 & 1 & 1 & 0 & 0 & 0 & 0 & 1 & 0 & 0 & 1 & 1 & 0 & 1\\ 0 & 0 & 0 & 1 & 1 & 1 & 0 & 0 & 1 & 0 & 0 & 1 & 0 & 1 & 0 & 0\\ 0 & 0 & 0 & 1 & 0 & 0 & 0 & 1 & 0 & 0 & 0 & 0 & 0 & 0 & 0 & 0\\ 1 & 1 & 1 & 0 & 1 & 0 & 0 & 0 & 0 & 1 & 0 & 0 & 0 & 0 & 1 & 0\\ 1 & 1 & 0 & 1 & 0 & 0 & 0 & 1 & 1 & 0 & 0 & 0 & 0 & 1 & 0 & 0\\ 0 & 1 & 0 & 0 & 0 & 0 & 0 & 1 & 1 & 1 & 0 & 0 & 1 & 0 & 0 & 1\\ 0 & 0 & 0 & 0 & 0 & 0 & 0 & 1 & 0 & 0 & 0 & 1 & 0 & 0 & 0 & 0\\ 0 & 0 & 1 & 0 & 1 & 1 & 1 & 0 & 1 & 0 & 0 & 0 & 0 & 1 & 0 & 0\\ 0 & 1 & 0 & 0 & 1 & 1 & 0 & 1 & 0 & 0 & 0 & 1 & 1 & 0 & 0 & 0\\ 1 & 0 & 0 & 1 & 0 & 1 & 0 & 0 & 0 & 0 & 0 & 1 & 1 & 1 & 0 & 0\\ 0 & 0 & 0 & 0 & 0 & 0 & 0 & 0 & 0 & 0 & 0 & 1 & 0 & 0 & 0 & 1\\ 0 & 1 & 0 & 0 & 0 & 0 & 1 & 0 & 1 & 1 & 1 & 0 & 1 & 0 & 0 & 0\\ 1 & 0 & 0 & 0 & 0 & 1 & 0 & 0 & 1 & 1 & 0 & 1 & 0 & 0 & 0 & 1\\ 1 & 1 & 0 & 0 & 1 & 0 & 0 & 1 & 0 & 1 & 0 & 0 & 0 & 0 & 0 & 1\\ 0 & 0 & 0 & 1 & 0 & 0 & 0 & 0 & 0 & 0 & 0 & 0 & 0 & 0 & 0 & 1\\ 1 & 0 & 0 & 0 & 0 & 1 & 0 & 0 & 0 & 0 & 1 & 0 & 1 & 1 & 1 & 0 \end{array}\right] \end{array}$$ (2)

Based on the reward function and the cost function, the cost value of the traffic on that road can be calculated as *C*_*i*_(*i* = 1,2,…,16). The cost value of each road *C*_*i*_ is calculated as follows:

$$C_{i}=\sum_{i=1}^{16}(E[i][j])*P_{i}$$ (3)

With reward value and cost value, the loss value for each road over the current period can be calculated.


$$M=C_{i}-R_{i}$$ (4)

At the same time, to solve the situation that different routes have the same loss value, we introduce a constraint function *ϵ*_*n*_, Set the value of the initial constraint function for each road to 0. The value of the route constraint function that is not enabled in the nth dispatch is increased by 1, and the value of the enabled road constraint function is reset to 0. The rules for setting constraint functions are as follows.


$$\{\begin{array}{l} \varepsilon_{n}=0,\;i\;route\;is\;enabled\\ \varepsilon_{n}=\varepsilon_{n-1}+1,\;i\;route\;is\;not\;enabled \end{array}$$ (5)

Selecting the route with the minimum loss value *M* as the first path in the scheduling scheme.

### Iterative update to achieve dynamic scheduling of traffic lights

Upon selecting the first route, continuous iterations are performed based on the current traffic conditions. Traffic signal states for all directions are updated within fixed time intervals to achieve the optimization of traffic flow.

**Fig 5:**
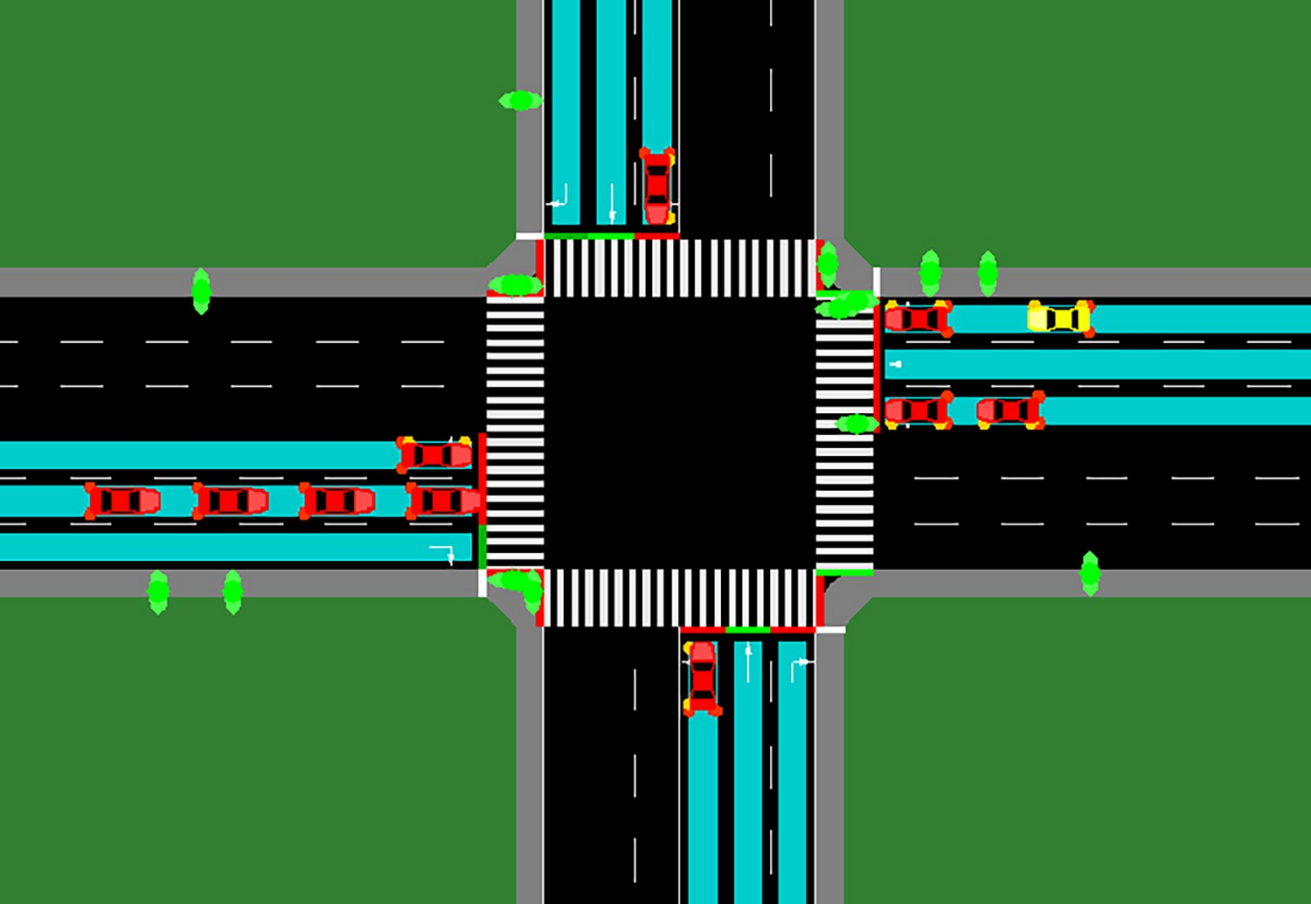
Congested intersection section.

As shown in the traffic section of Fig 5 above, after solving the first route using the greedy strategy, we proceed to solve the remaining routes. To facilitate this process, we transform the independent matrix A as follows to obtain the one-dimensional feasible array *F*:

$$\left\{ \begin{array}{l} S[i][j]=A[i][j](-1\to 0,1\to 1,0\to 0)\\ \Rightarrow S=\left[\begin{array}{cccccccccccccccc} 0 & 1 & 1 & 0 & 0 & 1 & 1 & 1 & 1 & 0 & 1 & 1 & 0 & 0 & 1 & 0\\ 1 & 0 & 1 & 0 & 0 & 0 & 1 & 1 & 0 & 1 & 1 & 0 & 1 & 0 & 1 & 1\\ 1 & 1 & 0 & 0 & 1 & 1 & 1 & 0 & 1 & 1 & 1 & 1 & 1 & 1 & 1 & 1\\ 0 & 0 & 0 & 0 & 0 & 1 & 1 & 1 & 1 & 0 & 1 & 1 & 1 & 1 & 0 & 1\\ 0 & 0 & 1 & 0 & 0 & 1 & 1 & 0 & 0 & 1 & 1 & 1 & 1 & 0 & 1 & 1\\ 1 & 0 & 1 & 1 & 1 & 0 & 1 & 0 & 0 & 0 & 1 & 1 & 0 & 1 & 1 & 0\\ 1 & 1 & 1 & 1 & 1 & 1 & 0 & 0 & 1 & 1 & 1 & 0 & 1 & 1 & 1 & 1\\ 1 & 1 & 0 & 1 & 0 & 0 & 0 & 0 & 0 & 1 & 1 & 1 & 1 & 0 & 1 & 1\\ 1 & 0 & 1 & 1 & 0 & 0 & 1 & 0 & 0 & 1 & 1 & 0 & 0 & 1 & 1 & 1\\ 0 & 1 & 1 & 0 & 1 & 0 & 1 & 1 & 1 & 0 & 1 & 0 & 0 & 0 & 1 & 1\\ 1 & 1 & 1 & 1 & 1 & 1 & 1 & 1 & 1 & 1 & 0 & 0 & 1 & 1 & 1 & 0\\ 1 & 0 & 1 & 1 & 1 & 1 & 0 & 1 & 0 & 0 & 0 & 0 & 0 & 1 & 1 & 1\\ 0 & 1 & 1 & 1 & 1 & 0 & 1 & 1 & 0 & 0 & 1 & 0 & 0 & 1 & 1 & 0\\ 0 & 0 & 1 & 1 & 0 & 1 & 1 & 0 & 1 & 0 & 1 & 1 & 1 & 0 & 1 & 0\\ 1 & 1 & 1 & 0 & 1 & 1 & 1 & 1 & 1 & 1 & 1 & 1 & 1 & 1 & 0 & 0\\ 0 & 1 & 1 & 1 & 1 & 0 & 1 & 1 & 1 & 1 & 0 & 1 & 0 & 0 & 0 & 0 \end{array}\right]\\ F_{i}=S[i] \end{array}\right.$$ (6)

According to *F* the calculated cost value:

$$\{\begin{array}{l} C_{j}=\sum_{i=1}^{16}(E[i][j])*P_{i}*F_{i}\\ M_{j}=C_{j}-R_{j} \end{array}$$ (7)

After finding the second route with the least loss value, it is necessary to iteratively update the variable *F* according to the following strategy for the nth iteration:

$$F=S[i]*S[j],\;\varepsilon_{n-1}\Rightarrow \varepsilon_{n},\;M_{n-1}\Rightarrow M_{n}$$

**Fig 6:**
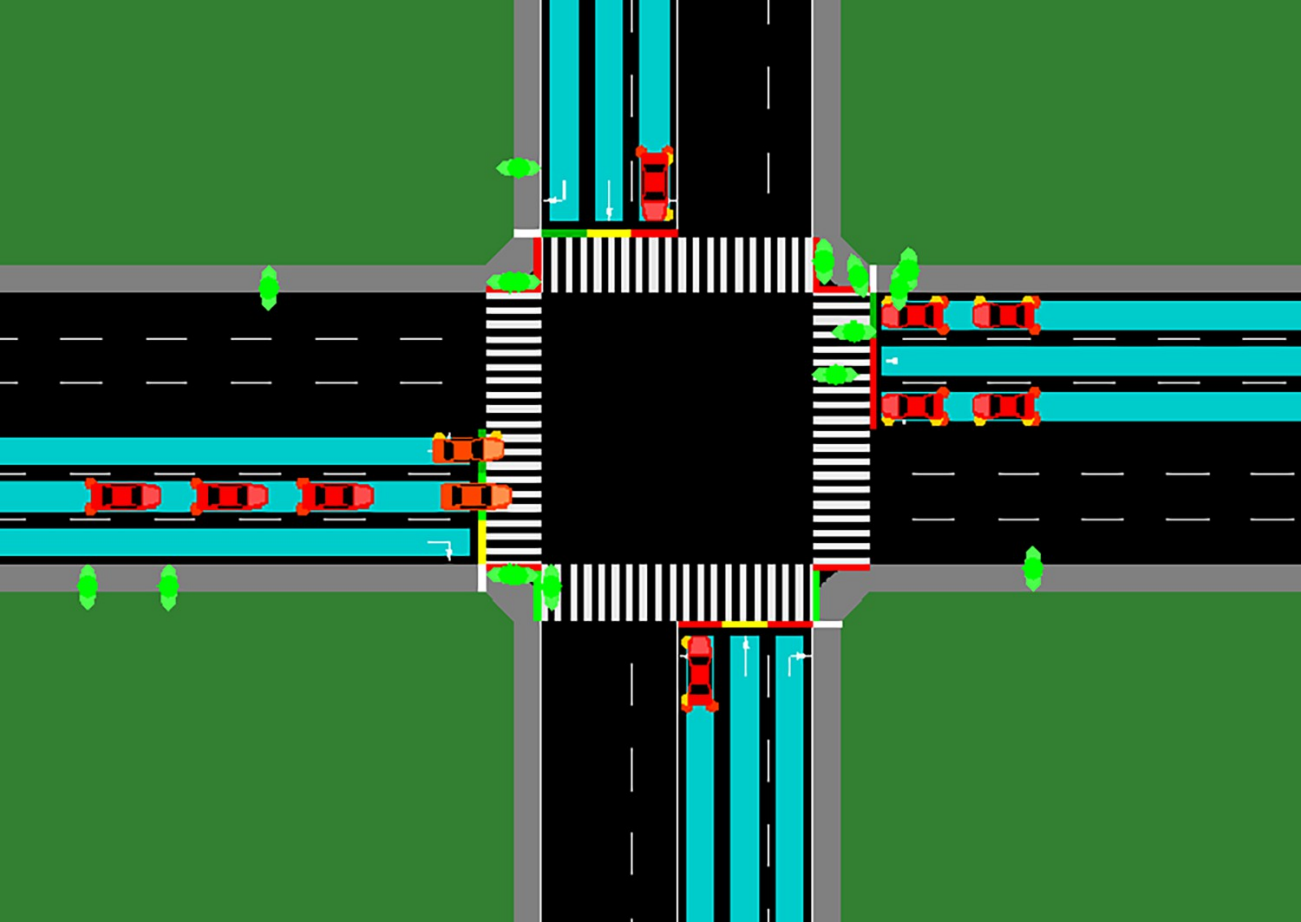
Crossroads after dispatching.

After iterating until the feasible array *F* is all 0, the scheduling is completed, and the optimal traffic flow is found. The traffic conditions after the dispatch are shown in Fig 6 below, demonstrating that the scheduling algorithm can effectively alleviate the traffic congestion problem.

### Emergency module

We propose the introduction of an emergency module in the traffic light dispatch system to prioritize the passage of special emergency vehicles, such as ambulances and police cars, thereby enhancing the efficiency and safety of the city’s transportation system.

In the dynamic signal dispatch system’s emergency module, our primary objective is to ensure traffic safety and prioritize public interest during emergencies. Different priorities can be assigned to various vehicles and pedestrians in the traffic scheduling algorithm. During emergencies, emergency service vehicles like police cars, ambulances, and fire engines are granted the highest priority to ensure the uninterrupted execution of their critical tasks. Consequently, we optimize the traffic scheduling mechanism based on the priority algorithm.

The vehicle type-based priority system is a traffic management approach that classifies vehicles according to type and assigns traffic priority accordingly. This method categorizes vehicles into two groups: emergency service vehicles and the rest of the vehicles. Emergency service vehicles, such as ambulances, police cars, and fire engines, are granted top priority due to their crucial role in responding to emergencies and ensuring rapid arrival at their destinations. The rest of the vehicles, excluding emergency service vehicles, are assigned the second priority to discourage private vehicle usage during peak periods.

***P***_***i***_ represents the priority of route ***i***; if an emergency vehicle is present on route *i*, ***P***_***i***_ = 1, otherwise, ***P***_***i***_ = 0. Mi represents the loss value on route *i* under normal scheduling. An emergency scheduling ***E***_***i***_ can be determined using a function:

$$E_{i}=\{\begin{array}{l} 0,\;P_{i}=0\\ 1,\;P_{i}=1\;and\;M_{i}\;is\;the\;\min imum \end{array}$$ (8)

In this function, the values of ***P***_***i***_ and ***M***_***i***_ determine the final emergency scheduling scheme ***E***_***i***_ **= 1** indicates that route *i* is designated as an emergency passageway, while ***E***_***i***_ **= 0** means route *i* is not designated as an emergency passageway. When an emergency vehicle is detected on the road, the emergency module is immediately activated, indicating ***P***_***i***_ = **1**, prioritizing the passage of route *i*. If multiple routes have ***P***_***i***_ values equal to 1, the loss values ***M***_***i***_ are compared, and the route *i* with the smallest Mi value is selected, setting ***E***_***i***_ **= 1** as the first emergency route. The process iterates, considering other routes with ***M***_***i***_ **= 1** until the feasible array F for routes with loss value ***M***_***i***_ **= 1** is all 0. Then, routes without emergency vehicles are iterated. When all initial routes have ***E***_***i***_ **= 0** indicating no emergency situation, the normal traffic light scheduling module is executed.

By considering both the priority (***P***_***i***_) and traffic route loss values (***M***_***i***_), the model effectively determines the emergency scheduling (***E***_***i***_) for the roads, enabling rapid adjustments in traffic flow during emergencies. Additionally, the model intelligently selects routes with the minimum loss values when multiple roads face emergency situations, progressively managing other routes to minimize traffic congestion and enhance efficiency. Its flexibility and adaptability are commendable, allowing adjustments according to changing traffic conditions. Furthermore, even in the absence of specific emergencies, the model seamlessly executes normal traffic light scheduling, ensuring the smooth operation of the traffic system.

### The improved greedy algorithm pseudocode

The improved greedy algorithm is implemented using the Python programming language and PyCharm software. The code is shown in Algorithm 1.

**Algorithm 1** The code of the improved greedy Algorithm

**Input:** emergency_vehicles_list, normal_vehicles_list, pedestrian_list, dispatch_interval

**Output:** Traffic Light Scheduling Scheme

1: **Function** TrafficLightScheduler(emergency_vehicles_list, normal_vehicles_list, pedestrian_list, dispatch_interval):

2: traffic_light_matrix = InitializeTrafficLightMatrix()

3: Loop Forever:

4:  selected_lights = []

5:  total_loss = CalculateTotalLoss(traffic_light_matrix)

6:  feasible_array = GenerateFeasibleArray(traffic_light_matrix)

7:  constraint_value = CalculateConstraintValue(traffic_light_matrix)

8:   has_emergency_vehicle = CheckForEmergencyVehicles(emergency_vehicles_list)

9:  **If** has_emergency_vehicle:

10:    HandleEmergencyVehiclePriority(traffic_light_matrix)

11:   Else:

12:    SelectNormalTrafficLights(traffic_light_matrix)

13:  feasible_array = UpdateFeasibleArray(feasible_array)

14:  **If** feasible_array is not empty:

15:   new_selected_lights = OptimizeLightSelection(feasible_array)

16:   selected_lights.extend(new_selected_lights)

17:  WaitFor(dispatch_interval)

18: End Loop

19: **Return** selected_lights

20: End Function

## Experiments and comparisons

To thoroughly validate the efficiency and superiority of our proposed scheduling system, we conducted several sets of experiments to evaluate its performance in various aspects. Our evaluation involved analyzing the performance of both the traditional signal system and the improved signal system under different configurations of traffic volumes and other attribute values. The key metrics we focused on during the evaluation included the average waiting time for vehicles and pedestrians, the standard deviation of waiting time, total fuel consumption, and gas emissions. These metrics allowed us to assess the effectiveness of the improved traffic system [43–45]. The experiments were divided into four parts, as outlined below.

To enhance the comprehensiveness of our evaluation and establish a comprehensive benchmark, we extended our analysis by incorporating a comparative study with a relatively advanced Deep Q-network (DQN)-based signal scheduling system [46]. In this comparative study, we considered the same sets of experiments conducted for the evaluation of our proposed system. Additionally, we integrated the DQN-based signal scheduling system into the experimental framework to gauge its performance across the same configurations of traffic volumes and attribute values.

### Scheduling frequency

**Fig 7:**
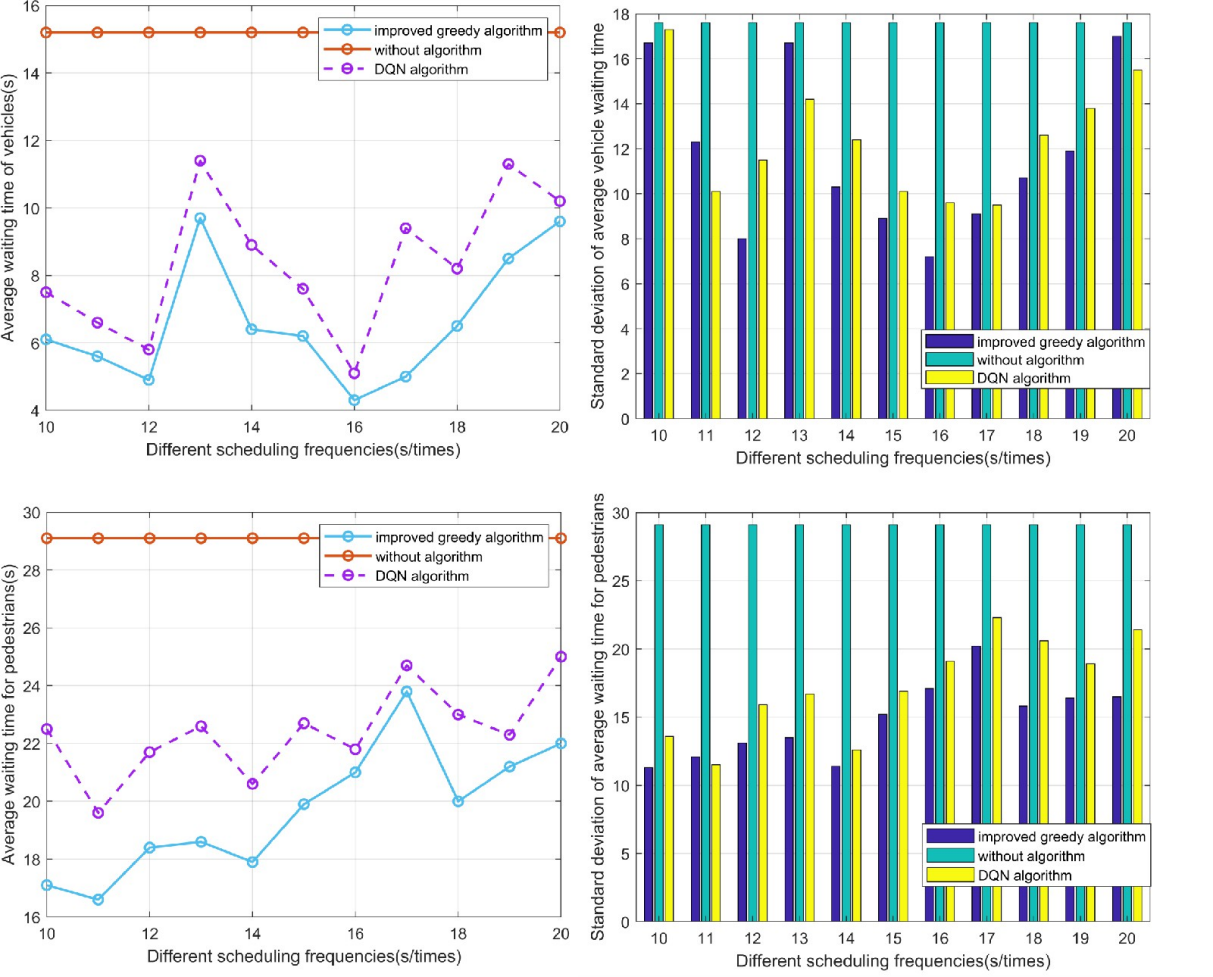
Average waiting time and standard deviation of vehicles (a) and pedestrians (b) before and after algorithms implementation.

Different dispatching frequencies can influence the efficiency of dispatching, thereby affecting the effectiveness of our improved traffic dispatching system. Therefore, we evaluated our traffic system by adjusting the dispatching frequency from 10 seconds to 20 seconds to change the signal once. The experimental results are shown in Fig 7.

From Fig 7, it is evident that within the dispatching time range of 10 to 20 seconds, the average vehicle waiting time shows a gradually increasing trend. However, the results of the dynamic signal dispatching system are significantly lower than the traditional results. Specifically, from Fig 7(A), the dynamic signal dispatching system reduces the waiting time by an average of 32.1%. Additionally, through the analysis of Fig 7(B), it is apparent that the dynamic signal dispatching system also yields a noteworthy improvement in pedestrian waiting times.

Furthermore, our dynamic signal dispatching system, incorporating an improved greedy algorithm, not only outperforms the traditional signal dispatching system but also demonstrates a comparative advantage over the signal dispatching system utilizing the DQN algorithm. It is noteworthy that, in the majority of cases, the standard deviations observed in waiting times for signal dispatching systems employing the DQN algorithm surpass those associated with our dynamic dispatch system implementing the improved greedy algorithm. This suggests a level of robustness and consistency in our proposed dynamic signal dispatching approach, indicating its potential advantages over alternative methods.

Considering real-life factors such as driver and pedestrian reaction times, vehicle acceleration, and pedestrian walking speeds, we recommend selecting dynamic traffic dispatching time of 18 seconds.

### Vehicle-to-pedestrian ratio

In the second experiment, we considered different traffic volumes and pedestrian flows at the intersection by changing the configuration of vehicles and pedestrians. We tested the adaptability of our dynamic traffic signal scheduling system under different traffic conditions. The scheduling time was set to 18s, the pedestrian count was set to 100, and the total simulation time was set to 400s. We set the vehicle count for three directions to 50 each and changed the number of vehicles in the other direction to change the vehicle-to-pedestrian ratio.

**Fig 8:**
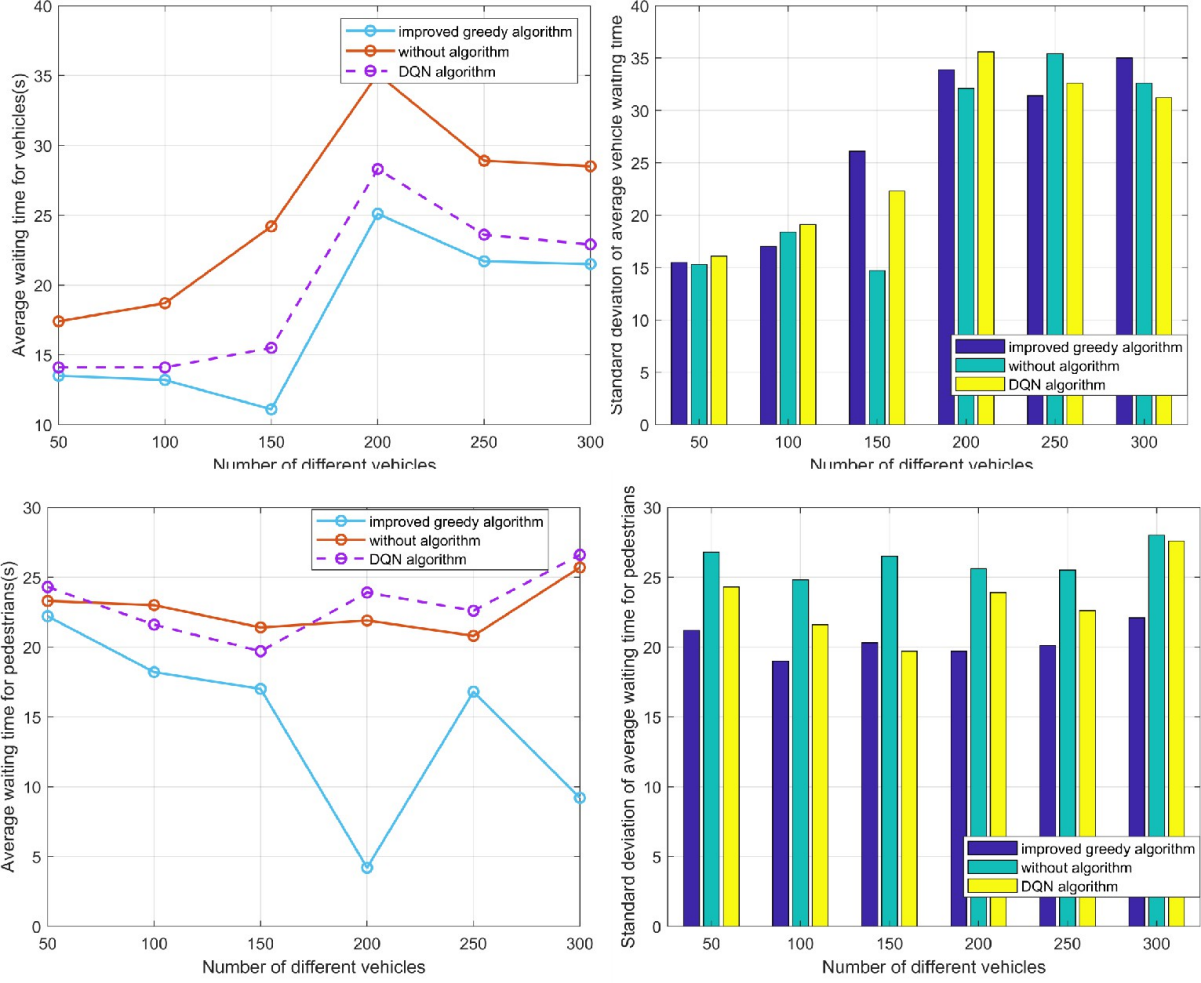
Average waiting time and standard deviation of vehicles (a) and pedestrians (b) with different vehicles.

Compared to traditional traffic signal systems, Fig 8 shows that our dynamic traffic signal scheduling system has strong optimization effects on different vehicle-to-pedestrian ratios. The average waiting times for vehicles and pedestrians in our dynamic system were significantly lower than those in traditional systems, and the standard deviations were generally lower as well. These results demonstrate that our dynamic traffic signal scheduling system is adaptable to different traffic conditions and can effectively handle the complex and varied traffic scenarios encountered in modern intersections.

In scenarios with increasing disparities in traffic volume, our dynamic signal dispatching system with an improved greedy algorithm outperforms the signal dispatching system with DQN algorithm. The waiting time for vehicles in our dynamic system with the improved greedy algorithm is notably less than that with the DQN algorithm, especially in extreme cases where the number of vehicles in one direction is 300. Moreover, the DQN algorithm lacks consideration for pedestrians, resulting in suboptimal waiting time data for pedestrian traffic.

### Fuel consumption and gas emissions

One of the primary objectives of traffic scheduling systems is to optimize traffic flow, reduce traffic congestion, improve road utilization, and minimize unnecessary fuel consumption and gas emissions. Therefore, in the third experiment, we utilized fuel consumption and gas emissions as indicators to compare the total fuel consumption and gas emissions of traditional traffic systems with our dynamic traffic signal scheduling system. By varying the configuration of vehicle counts and conducting traffic simulations, we evaluated the system’s performance.

**Fig 9:**
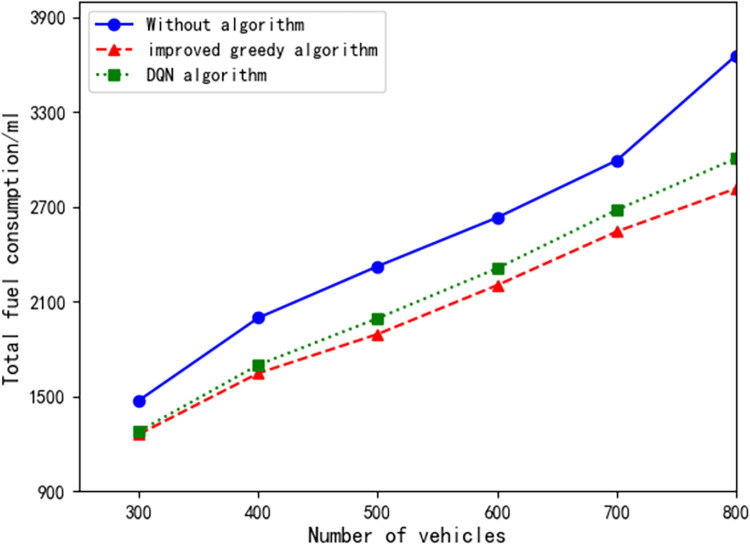
Total fuel consumption under different vehicle configurations.

**Fig 10:**
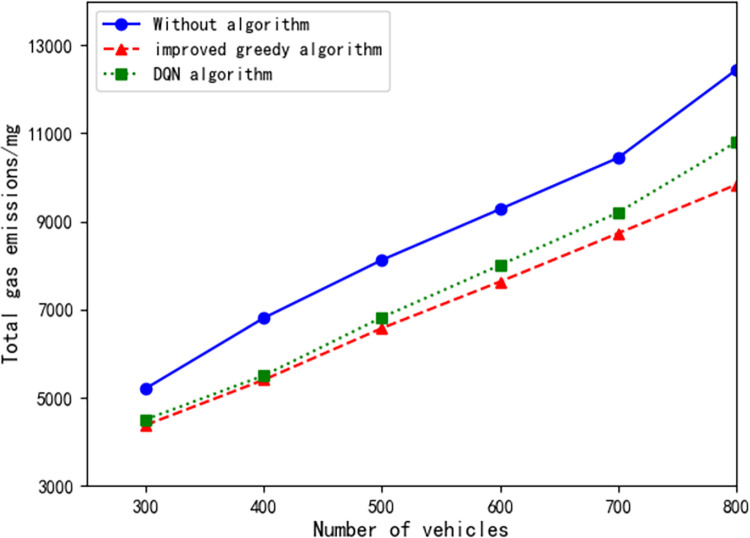
Gas emissions under different vehicle configurations.

As shown in Figs 9 and 10, our dynamic signal dispatch system demonstrates remarkable efficiency in reducing vehicle fuel consumption at signal road intersections. Compared to the traditional transportation system, Table 2 reveals that, in the studied scenario, the dynamic signal light dispatching system reduced the total fuel consumption by an average of 17.481% and gas emissions by an average of 18.456%. Additionally, when compared to a scheduling system utilizing DQN algorithm, our dynamic signal light dispatching system achieved a further average reduction of 4.275% in total fuel consumption and 4.482% in gas emissions (see Table 2). These findings demonstrate that the dynamic signal light dispatching system significantly reduces fuel consumption and emissions. The system optimizes vehicle travel at intersections, reduces vehicle wait times, and minimizes instances of signal lights turning on in vain, thus leading to reduced fuel consumption and gas emissions.

**Table 2:** Fuel consumption and emission rates are reduced under different vehicle configurations.

Number of vehicles	Fuel consumption reduction rate compared to conventional	Fuel consumption reduction rate compared to DQN	Gas emission reduction rate compared to conventional	Gas Emission Reduction Rate compared to DQN
300	14.529%	1.470%	15.950%	2.779%
400	17.549%	3.091%	20.640%	1.778%
500	18.485%	5.030%	19.057%	3.578%
600	16.274%	4.638%	17.735%	4.694%
700	15.032%	5.114%	16.401%	5.108%
800	23.020%	6.307%	20.951%	8.955%
Average reduction rate	17.481%	4.275%	18.456%	4.482%

### Number of special emergency vehicles

**Fig 11:**
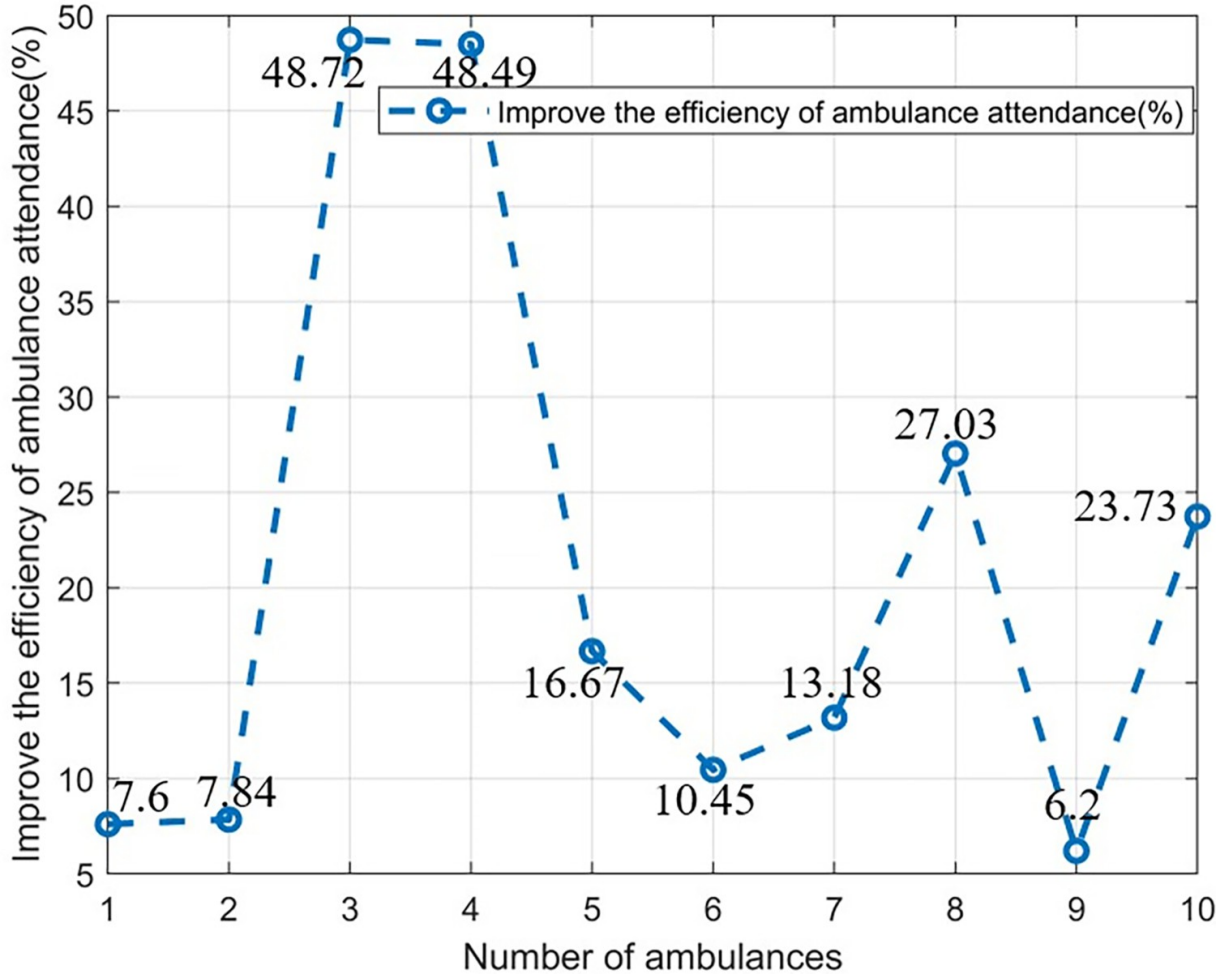
Attendance efficiency under different ambulance configurations.

We evaluate the emergency module of the improved dynamic traffic light dispatch system, considering different special emergency vehicle configurations (using ambulances as an example) to verify the superiority of our improved system in various emergencies. Because there may be two special emergency vehicles at the same intersection, how to emergency dispatch is particularly critical. The experimental results are shown in Table 3 and Fig 11.

**Table 3:** Traffic dispatch under different ambulance configurations.

Number of ambulances	Average waiting time for ambulances not using a dispatch system	Average waiting time for ambulances using a dispatch system	Improve the efficiency of ambulance attendance
1	2.50	2.31	7.600%
2	3.19	2.94	7.837%
3	7.41	3.80	48.718%
4	8.25	4.25	48.485%
5	7.20	6.00	16.667%
6	7.85	7.03	10.446%
7	9.71	8.43	13.182%
8	7.88	5.75	27.030%
9	6.78	6.36	6.195%
10	6.70	5.11	23.731%

Here we set the scheduling time to 18 seconds. We can see that for special emergency vehicles with different configurations, the average waiting time of special vehicles with our emergency algorithm is significantly lower than that of traditional time scheduling methods, and our emergency module can reduce the average waiting time of special emergency vehicles by 27.8%.

Therefore, our emergency modules are proven to be effective in various traffic situations, as evidenced by the reduced waiting time for special emergency vehicles, even when different configurations are considered.

## Outlook and future work

Although the simulation process takes real-life situations into full account, there are always inevitable errors in real-world scenarios that may not align perfectly with the data we simulate.

Currently, computer vision detection technology is not perfect, and there is a certain degree of error in detecting the number of vehicles. In our simulated SUMO environment, we assume that the data recognized by the camera is unbiased, ensuring that the simulated traffic conditions accurately represent real-world scenarios. Therefore, further research is needed to improve the accuracy of visual detection for vehicle identification. Additionally, introducing traffic flow prediction can help reduce the impact of data noise on traffic scheduling and enhance the robustness of signal light scheduling systems.

The scheduling of traffic signals at a single intersection has limitations after all, such as suboptimal coordination with nearby intersections. In the future, we will extend the dynamic signal scheduling algorithm based on an improved greedy algorithm from a single intersection to a region. To overcome these limitations, we aim to extend the dynamic signal scheduling algorithm from a single intersection to a regional level. This involves generating a complex global traffic network through signal transmission, unified scheduling, and dynamic traffic planning across multiple intersections. Strengthening the connection between intersections will lead to significant reductions in travel time and improved comfort for vehicle travel.

### Summary

In conclusion, urban traffic congestion can be alleviated by controlling traffic signals, but traffic scheduling has always been a challenging problem. Unlike other traffic scheduling systems, this article proposes a dynamic signal control system based on an improved greedy algorithm, which changes the fixed sequence to solve the traffic signal scheduling problem and fully considers the situation of special emergency vehicle dispatch.

We utilize the greedy algorithm to decompose the problem of optimizing traffic flow into several periods, enabling efficient and effective decisions for each scheduling interval. Our method introduces reward functions, cost functions, and constraint functions in each period to ensure the feasibility of the scheduling plan. Through iterative updates based on the current number of vehicles and pedestrians on the road, we obtain the final scheduling plan. Additionally, the emergency module prioritizes emergency special vehicles and uses the improved greedy algorithm to iteratively update and select the best emergency scheduling strategy.

Our simulation experiments using the SUMO traffic simulation software focus on four key aspects for evaluation: scheduling frequency, the ratio of vehicles and pedestrians, fuel consumption and gas emissions, and the number of special emergency vehicles. The thorough data analysis demonstrates the effectiveness of our proposed algorithm in addressing various traffic conditions at intersections and catering to special emergency vehicle dispatch scenarios.

In summary, our algorithm demonstrates effective performance in solving the traffic signal scheduling problem under various traffic conditions at intersections while efficiently addressing the needs of special emergency vehicle dispatch.

## Supporting information

S1 Data (XLSX)
